# Dr. William Moxon

**Published:** 1911-08-12

**Authors:** 


Dr. William Moxon, of Matlock, Derbyshire, has died aged fifty-seven. He studied at Queen's College, Birmingham, and took his M.R.C.S. and L.R.C.P. in 1876, and obtained his M.D. degree at the University of Durham in 1895. His appointments both public and professional included those of consulting physician to the Hydropathic Hospital and the Derbyshire Home, at Matlock, chairman of the Whitworth Hospital Board, physician to the Matlock House Sanatorium, and medical officer of health for Matlock. Dr. Moxon was known in various social activities at Matlock and took active interest in the old Volunteer movement.

